# The health gains and cost savings of dietary salt reduction interventions, with equity and age distributional aspects

**DOI:** 10.1186/s12889-016-3102-1

**Published:** 2016-05-23

**Authors:** Nhung Nghiem, Tony Blakely, Linda J. Cobiac, Christine L. Cleghorn, Nick Wilson

**Affiliations:** University of Otago, PO Box 7343, Wellington, Wellington South New Zealand; British Heart Foundation Centre on Population Approaches to NCD Prevention, Oxford University, Oxford, UK

**Keywords:** Sodium, Dietary salt, Cardiovascular disease, Salt substitution, Bread, Modelling

## Abstract

**Background:**

A “diet high in sodium” is the second most important dietary risk factor for health loss identified in the Global Burden of Disease Study 2013. We therefore aimed to model health gains and costs (savings) of salt reduction interventions related to salt substitution and maximum levels in bread, including by ethnicity and age. We also ranked these four interventions compared to eight other modelled interventions.

**Methods:**

A Markov macro-simulation model was used to estimate QALYs gained and net health system costs for four dietary sodium reduction interventions, discounted at 3 % per annum. The setting was New Zealand (NZ) (2.3 million adults, aged 35+ years) which has detailed individual-level administrative cost data.

**Results:**

The health gain was greatest for an intervention where most (59 %) of the sodium in processed foods was replaced by potassium and magnesium salts. This intervention gained 294,000 QALYs over the remaining lifetime of the cohort (95 % UI: 238,000 to 359,000; 0.13 QALY per 35+ year old). Such salt substitution also produced the highest net cost-savings of NZ$ 1.5 billion (US$ 1.0 billion) (95 % UI: NZ$ 1.1 to 2.0 billion). All interventions generated relatively larger per capita QALYs for men vs women and for the indigenous Māori population vs non-Māori (e.g., 0.16 vs 0.12 QALYs per adult for the 59 % salt substitution intervention). Of relevance to workforce productivity, in the first 10 years post-intervention, 22 % of the QALY gain was among those aged <65 years (and 37 % for those aged <70).

**Conclusions:**

The benefits are consistent with the international literature, with large health gains and cost savings possible from some, but not all, sodium reduction interventions. Health gain appears likely to occur among working-age adults and all interventions contributed to reducing health inequalities.

**Electronic supplementary material:**

The online version of this article (doi:10.1186/s12889-016-3102-1) contains supplementary material, which is available to authorized users.

## Background

A diet high in sodium is ranked as the second most important dietary risk factor to health globally according to the Global Burden of Disease Study 2013 [1]. The scale of this problem has resulted in “salt reduction” being included in the top five priority actions for non-communicable disease (NCD) control internationally [2] and for reducing NCD inequalities [3]. The World Health Organization (WHO) also recommends a “reduction to <2 g/day sodium (5 g/day salt) in adults (strong recommendation)” [4].

We have discussed elsewhere, the persisting concerns that salt reduction is not (as) beneficial as claimed [5]. Nevertheless, the totality of the evidence appears strong enough to justify public health action (i.e., the evidence from systematic reviews [6, 7] and specifically the data from various key trials [8–10]). Indeed, such justification is strengthened by more recent evidence that includes: a 2015 review [11]; a systematic review indicating CVD risk reduction benefits of the DASH (low sodium) diet [12]; and a new systematic review on salt and increased CVD mortality [13]. Even when the theoretical minimum level of risk exposure of sodium intake is modelled with very wide uncertainty (from 1000 to 5000 mg of dietary sodium per day), the Global Burden of Disease 2013 Study still found that dietary sodium was ranked as the eighth most important risk factor for health loss for developed countries [1].

There are a number of modelling studies which have considered the health gain and associated costs implications of dietary sodium reduction. The 10 studies we identified that used a health metric of quality-adjusted life-years (QALYs) or disability-adjusted life-years (DALYs) are shown in Table 1. This table also highlights some of the limitations with this literature including a lack of detail as to the specific details of the salt reduction intervention/s and usually only a small number of interventions being compared per model. Because modelling assumptions vary, so that interventions from one study often cannot be compared to interventions from another study, the range of choices for policy-makers interested in sodium reduction is still fairly constrained.

**Table 1:** Health economic modelling studies of population-level dietary salt reduction interventions in high-income countries which use the health metric of QALYs or DALYs and have cost implications (ordered by publication date^a^)

Setting and reference	Interventions	Main results	Comment e.g., on key features and potential limitations
26 European countries^b^, Murray et al 2003 [52]	(i) Cooperation between government and the food industry for a stepwise decrease in salt content of processed foods and for labelling; (ii) Legislation to decrease sodium content in processed foods and appropriate labelling (a combined package); (iii) Health education focusing on body mass index and cholesterol concentrations; (iv) A combined package of (ii) and (iii).	0.7-1.3 million DALYs averted per year (in European countries group). Very cost-effective at US$14-37 per DALY averted (DR = 3 %). Legislation reported to be more cost-effective than voluntary agreements.	This study did not consider cost-savings from preventing CVD. It was reliant on the relatively simplistic WHO Choice methodology for costing intervention programmes.
US, Palar & Sturm 2009 [53]	Reducing average population sodium intake to 1200-2300 mg/day [d].	Large annual QALY gains (312,000) and large annual savings in health costs (US$18 billion).	QALYs were also valued as part of a societal perspective. No specific intervention or intervention costs.
US, Smith-Spangler et al 2010 [54]	(i) Collaboration with industry to reduce sodium by 9.5 %, and (ii) a sodium tax to reduce sodium by 6 %.	Both interventions achieved large QALY gains (2.1 million and 1.3 million respectively over the cohort’s lifetime). Cost-savings at $US32.1 and 22.4 billion respectively.	A high quality study but the cost of implementing the tax intervention was not considered.
US, Bibbins-Domingo et al 2010 [18]	A regulatory intervention to reduce the level of salt intake by 3 g/d.	Large annual QALY gains (194,000 to 392,000) and annual cost-savings at $US10 to 24 billion. Salt reduction was more cost-effective than treating hypertension with medications. The anticipated relative benefits in blacks were greater than those for non-blacks across all age and sex groups.	A high quality study but no specific intervention or intervention costs detailed. This is only one of two studies in this table to consider ethnic inequalities.
Australia, Cobiac et al 2010 [55]	Voluntary and mandatory reduction of salt content in breads, margarine, and cereals; dietary advice; and labelling programme.	Both salt content interventions were cost-saving (e.g., $A3.3 billion for the mandatory one over the cohorts lifetime) but health gain was much greater for the mandatory vs voluntary intervention (e.g., 110,000 vs 5300 lifetime DALYs averted). The labelling programme was cost-effective but not the dietary advice.	Included a useful comparison between a voluntary and mandatory intervention. Used WHO Choice methods rather than more country-specific intervention costing data.
England & Wales, Barton et al 2011 [56]	Legislative means (unspecified) to reduce salt intake by 3 g/d	Any salt-reduction intervention costing up to £40 million a year was estimated to be cost-saving. For a 3 g/d reduction over 10 years the total QALY gain was 131,000.	No specific intervention was modelled. See also comments in a review [57].
Finland, Martikainen et al 2011 [17]	A population-wide 1 g/d salt reduction (by unspecified means).	Large QALY gains (26,100 by the year 2030). Cost-savings were 150–225 million Euros by 2030 (but when combined with the saturated fat reduction intervention).	Also considered reductions in productivity losses. No specific intervention was modelled. See also comments in a review [57].
Australia, Cobiac et al 2012 [58]	Mandatory reduction of salt content in breads, margarine, and cereals; and Community Heart Health Programme (CHHP).	Large number of DALYs averted per year (80,000) for the mandatory intervention (vs 3000 in the CHHP) and cost-saving. (See also a similar study listed above by these authors).	This study allowed for a comparison of the mandatory salt reduction with various CVD treatment interventions (the former being more cost-effective).
England, Dodhia et al 2012 [59]	Included: (i) reductions of salt leading to 2 mmHg and (ii) 5 mmHg reductions in blood pressure; (iii) reduced intake down to 6 g/d via assumed food industry agreement; (iv) advice for DASH-sodium diets.	Large number of DALYs averted for (i) to (iv) in the 200,000 to 900,000 range (DR = 3.5 %). Salt reduction in the population was always reported to be cost-saving except for dietary advice in some age-groups (but here it was still cost-effective). The maximum saving for an intervention was £1.9 billion (over 10y).	High quality study which allowed comparisons with CVD treatment interventions.
New Zealand, Nghiem et al 2015 [5]	Eight interventions (mix of mandatory and voluntary interventions – see Table 5 for findings combined with the results of this particular study).	All interventions (except dietary counselling) were cost-saving. The largest gain was from a “sinking lid” intervention (211,000 QALYs over the cohort’s lifetime; $US0.7 billion in savings). The interventions were estimated to produce relatively greater health gain for indigenous people (Māori).	The study had some limitations including around price elasticity data (for the salt tax) and the hypothetical nature of some interventions (e.g., sinking lid). See the Discussion where we compare these results with the current analysis.

^a^The literature search period in PubMed was from undated to the end of July 2015. The search terms were “sodium or salt” and “QALYs or DALYs”. Of the identified studies, some were screened out since they lacked any data on cost implications or were not for high-income countries

^b^Countries covered in the European “A” region: Andorra, Austria, Belgium, Croatia, Czech Republic, Denmark, Finland, France, Germany, Greece, Iceland, Ireland, Israel, Italy, Luxembourg, Malta, Monaco, Netherlands, Norway, Portugal, San Marino, Slovenia, Spain, Sweden, Switzerland, UK

Given the relatively limited range of such existing salt reduction interventions modelled to date, we aimed to use an existing model [5] to consider additional interventions. We selected salt substitution as one intervention area in that it has not been modelled before for cost-effectiveness (to our knowledge) and yet is an intervention area with major potential [14–16]. To provide a contrast and also to model what might be a first step for policy-makers considering salt reduction, we also modelled feasible interventions around setting maximum levels of sodium in bread (a relatively important sodium source in many countries).

The studies in Table 1 also suggest the limited work on the impacts of salt reduction interventions on workforce productivity (just the one Finnish study [17]) and health inequalities (just two studies [18] [5]). Hence we also aimed to also consider the distribution of the health gain by sex, age and ethnic groups.

## Methods

### Model structure and perspective

As described previously [5], we used a Markov macro-simulation model in TreeAge Pro version 2013. The simulated population was a closed cohort of the New Zealand population aged 35 years and older (2.3 million people), modelled from the baseline year (2011) to death or age 100 years. The Markov model has four primary health states, with annual transition rates capturing incidence and case-fatality for coronary heart disease (CHD) and stroke events (see the diagram in an online Technical Report [19]). Essentially, proportions of each age/sex/ethnicity cohort occupy the states of: being “healthy” (i.e., not having CVD), having a form of CVD (CHD or a type of stroke), or death, in each annual cycle.

In terms of modelling background disease trends we took the same approach as the New Zealand Burden of Disease Study (NZBDS) [20], and assumed a continued decline in incidence rates for both CHD and stroke of 2.0 % annually, and also a 2.0 % reduction in case-fatality annually (i.e., reflecting improved treatment and management, and resulting in 4 % per annum decline in mortality when combined with incidence trends). We extended this projection from 2016 (NZBDS end estimate) to the year 2026 and then held the incidence and case-fatality rates constant. Nevertheless, background population mortality was assumed to decline at a somewhat lower rate than for CVD with a 1.75 % annual reduction for non-Māori, and 2.25 % [21] for the indigenous population of Māori (also out to the year 2026), then 0 % per annum decline for both ethnic groupings thereafter.

A health system perspective was used with costs and benefits beyond the health system (e.g., productivity gains from preventing premature deaths of workers) being considered out of scope. However, additional health system costs arising from extra life expectancy in the future attributable to the impact of the modelled interventions were included in the baseline analyses. Costs were calculated in 2011 New Zealand (NZ) dollars and a 3 % discount rate was applied to costs and future health gain. OECD 2011 purchasing power parities [22] were used for calculating results in US$ for comparative purposes.

All interventions were evaluated against a theoretical “do nothing” comparator (i.e., doing none of the interventions of interest in the analysis) [23]. Therefore, we back-calculated disease rates under the “do nothing” scenario using the same parameters of intervention effectiveness, adherence and costs that are used in the cost-effectiveness analyses. This required us to remove the impact of the sodium reduction interventions currently in place in New Zealand in the baseline year i.e., dietary counselling by dietitians and an endorsement label programme [5].

### Input parameters

Baseline input parameters are shown in Table 2 and further background is given in the following text.

**Table 2:** Main input parameters to the modelling: selected baseline and epidemiological parameters

Variable	Sources and key details	Key values and uncertainty
Baseline variables in 2011		
Sodium intake	Source: New Zealand (NZ) nutrition survey data [60], with significant variation by sex, but not by ethnicity or age (for adults). No trend under business-as-usual (BAU) specified, given no notable trend since the 1980s [61]. Although these values are based on spot urine tests, such tests are a reasonable means for studying populations as per this systematic review [62]. The values are also similar to previous NZ studies which have used 24-h urine collections [63].	4013 mg/d for men and 3115 mg/d for women (nil uncertainty; rather uncertainty around the intervention associated reduction was considered – see below)
Incidence, prevalence and case-fatality data for CHD and stroke	Calculated using linked Health Tracker data, with coherency checks using DisMod II and smoothing with regression as required. Future annual percentage change (APC) in incidence and CFR were both set at -2.0 % each as per the NZBDS.	See online reports for details [19, 32].
Morbidity (disability weights [DW])	From GBD2010 [25], with modification to NZ [20] and slight variation by age and ethnicity (see an online report [19] for details).	DW for CHD = 0.081 (average) DW for stroke = 0.226 (average). Uncertainty: e.g., for non-Māori males, 95%CI: 0.05–0.11 for CHD and 0.11–0.23 for stroke. (For more details uncertainty see Nghiem et al [19]).
Baseline health system costs for CHD and stroke states, and non-diseased states	Calculated from Health Tracker data by sex and age in 2011 for people: (a) without either CHD or stroke; (b) with CHD only, and excess to (a); (c) with stroke only, and excess to (a). (See an online report [19] for details).	Examples for 60 year old women (gamma distribution with SD = 10 % of mean): (a) NZ$2381; (b) NZ$16,258 for the first year, NZ$5,395 for second and subsequent years; (c) NZ$20,553 and NZ$5991 for stroke.
Epidemiological associations		
Change in systolic blood pressure (sBP) (in mm Hg) for each 100 mmol/d change in sodium intake	Derived from the regressions models developed by Law et al [35]. The small differences in BP by ethnic group did not justify separate modelling by ethnicity (higher in Māori by 3 mm Hg for systolic BP and 4 mm Hg for diastolic BP in both sexes compared to non-Māori [64]). Also of note is that no trend in BP into the future was considered given the unclear picture in NZ (of a downward trend in population BP levels from 1982 to 2002 and then an upward trend from then 2008/09) [64].	For men and women:
		Age-group; sBP (mm Hg) change
		30–39: 5.5
		40–49: 6.6
		50–59: 9.2
		60–69: 10.3
Relationship between blood pressure and CVD risks	We used the results of a meta-analysis of 61 prospective studies by Lewington et al [36]. These results were considered to be more generalisable to the general population than those from a meta-analysis by Law et al 2009 of 147 RCTs of blood pressure-lowering drugs [65].	The hazard ratio for a 20 mm Hg reduction in systolic BP ranged from 0.49 to 0.67 for CHD and from 0.38 to 0.67 for stroke (depending on age). For uncertainty: SD = +/- 10 % of the point estimate for each age group.

#### Incidence, prevalence and case-fatality

The estimated incidence, prevalence and case-fatality rates of CHD and stroke (ischemic and haemorrhagic) were calculated across all combinations of sex, age-group (35–39, 40–44, … 95+ years) and ethnicity (Māori; and non-Māori). Data came from Ministry of Health data, called “Health Tracker”, a collection of linked administrative datasets of publically-funded health system events [24]. This includes hospitalisations, mortality, cancer registrations, mental health and addiction service use, pharmaceutical and laboratory claims, primary health care enrolment, and outpatient/emergency department visits for the entire New Zealand population with costs attached.

#### Morbidity and disability weights

Overall morbidity, by sex, age and ethnicity, was quantified in the model using the years of life lived with disability (YLDs) from the NZBDS [20], divided by the population count to give ‘prevalent’ YLDs. Disease-specific morbidity was assigned in each disease state (e.g., CHD and stroke), as the total comorbidity-adjusted YLDs for that disease divided by the prevalent population. The health status valuation used to calculate these YLDs were disability weights (DW) derived from the Global Burden of Disease study (GBD2010) using pair-wise comparisons from multi-country surveys [25]. These DWs are on a scale from 0 (full health) to 1.0 (death)-and included uncertainty (for details see the online Technical Report [19]). As per other BODE^3^ Programme work, we assumed no future underlying trend in morbidity burdens (i.e., both the size of the DWs and the background level of non-CVD morbidity were assumed constant into the future though we note complexities with interpreting recent New Zealand trends in morbidity [26]).

#### Intervention specification and parameters

We considered four additional interventions to our previous work [5]. These related to replacing some of the sodium chloride in all processed foods with other salts (at two different replacement levels, 25 and 59 %), and around maximum levels of sodium permitted in commercial bread (280 mg and 400 mg/100 g). Although described in further detail in Table 3, the key features were as follows:

**Table 3:** Input parameters relating to the four new intervention effects

Intervention	Sources and extra details	Key values and uncertainty (average adult)^a^
Salt substitution at 59 %: In all processed food the NaCl is legally required to be largely substituted with other salts at the level of 59 % (mix of potassium and magnesium salts).	This 59 % substitution level was that used in a randomised trial in the Netherlands [27]. This trial reported that all the foods were rated equally by both groups for appearance and palatability-except that the bread and table salt were considered “less salty” by those in the intervention group. It was assumed that the intervention would apply to processed foods (72 % of sodium intake in NZ) and table salt (15 % of intake), but not to milk, seafood, fruit, vegetables and fresh meat (13 % of intake) [66]. Of note, is that another study found that consumption of bread was not affected at 52 % reduction in sodium and up to 67 % when KCl and yeast extract was used for flavour compensation [46]. We assumed a phased-in implementation over a five year period (i.e., five equal steps beginning in the baseline year of 2011).	Reduction of 51.5 % in daily sodium intake from the reduced intake of processed foods and table salt (or 1824 mg [79.3 mmol] sodium per day for an average adult). For uncertainty we used +/- 10 % of the point estimate (normal distribution).
Salt substitution at 25 %: As per the intervention above but at a lower level.	This substitution level is as per the majority of the salt substitution studies in the meta-analysis by Peng et al [16]. The same assumptions about the phase-in period and application to just processed foods and to table salt (as per the intervention directly above) applied.	Reduction of 21.8 % in daily sodium intake from the reduced intake of processed foods and table salt (or 773 mg [33.6 mmol] sodium per day for an average adult). For uncertainty we used +/- 10 % of the point estimate (normal distribution).
Tight limits on sodium in bread: A legal requirement for commercial bread to have a sodium level that is ≤280 mg/100 g.	This is the level used in Finnish law for the labelling of low salt bread (i.e., 0.7 % salt) [28]. This level is equivalent to 0.28 % sodium which is equivalent to 0.28 g (or 280 mg) sodium per 100 g. For the baseline year we used the mean value of 439 mg/100 g for NZ bread [29]. We also assumed that to ensure ready compliance with the law, the manufacturers aim for an average level in bread that is slightly lower at 270 mg per 100 g. So this would shift the contribution of dietary sodium from bread from 20.6 % [66] to 12.7 % (270/439 x 20.6), i.e., a 7.9 % absolute reduction. That is a reduction of 280 mg/day (out of the baseline consumption of 3544 mg/day [60]). Of note is that this level is still higher than some breads currently on the NZ market e.g., 186 mg/100 g for one multigrain bread [30]. We assumed a phased-in implementation over a five year period (i.e., five equal steps beginning in the baseline year of 2011).	Reduction of 7.9 % (or 280 mg [12.2 mmol] sodium per day for an average adult). For uncertainty we used +/- 10 % of the point estimate (normal distribution).
Modest limits on sodium in bread: As per the intervention above but for a less stringent limit of ≤400 mg/100 g (i.e., as per a target value for Australia [31])	As per the row above except that we assumed that to ensure ready compliance with the law, the manufacturers aimed for the 390 mg per 100 g level. So this would shift the dietary contribution of sodium from bread from 20.6 to 18.3 % (390/439 × 20.6), i.e., a 2.3 % absolute reduction. That is a reduction of 81.5 mg/day (out of the baseline consumption of 3544 mg/day). We assumed full implementation in the baseline year.	Reduction of 2.3 % (or 81.5 mg [3.5 mmol] sodium per day for an average adult). For uncertainty we used +/- 10 % of the point estimate (normal distribution).

^a^Values given for the average adult. In the modelling we adjusted these values for men and women by ratios of 4013/3544 and 3115/3544 respectively, given the variation in sodium intakes (in mg) according to national nutrition survey data [60]

- Salt substitution at 59 % was at a level that was successfully used in a randomised trial in the Netherlands [27]. For the New Zealand population this was estimated to involve a reduction of 51.5 % in daily sodium intake from the reduced intake of processed foods and table salt (or 1824 mg [79.3 mmol] sodium per day for an average adult).
- Salt substitution at 25 % was at a level used in the majority of the salt substitution studies in a meta-analysis [16]. This was estimated to involve a reduction of 21.8 % in daily sodium intake from the reduced intake of processed foods and table salt (or 773 mg [33.6 mmol] sodium per day for an average adult).
- Tight limits on sodium in bread (≤280 mg/100 g) were based on the level used in Finnish law for the labelling of low salt bread (i.e., 0.7 % salt) [28]. This was estimated to involve a reduction of 7.9 % (or 280 mg [12.2 mmol] sodium per day for an average adult). For the baseline year, we used the estimated mean value of sodium in New Zealand bread of 439 mg/100 g [29], and we note that some commercial breads are already well below the intervention level (e.g., 186 mg/100 g for one multigrain bread [30]).
- Modest limits on sodium in bread (of ≤400 mg/100 g) were based on a target value for Australia [31]. This was estimated to involve a reduction of 2.3 % (or 81.5 mg [3.5 mmol] sodium per day for an average adult).

#### Costing of intervention scenarios and health system costs

We considered the net cost, which is the intervention costs plus health system costs throughout the lifespan of the modelled cohort (i.e., the results captured additional health costs associated with any extra lifespan generated by the interventions). Specific details for the costing of the interventions are provided in Table 4. For health system costs, the ‘business-as-usual’ ones were determined by strata of sex and age using Health Tracker data, which links cost estimates to all health events. From this dataset we calculated the 2011 costs for the first year of CHD and stroke, and then the average annual cost for the second and subsequent years. Furthermore, given that CVD is a relatively important part of baseline health system costs, we adjusted the baseline health system costs experienced by the “healthy” component of the modelled population, to remove the CVD-attributable cost component (to avoid double-counting).

**Table 4:** Input parameters relating to the interventions costs

Intervention	Intervention costs
Salt substitution at 59 %	The cost was that of a new law for NZ, which was based on the average cost of new act [67] at NZ$ 3,680,000 in 2011 dollars (these NZ$ dollar values are detailed in an online report [68]). Salt substitutes can cost around 50 % more than normal salt [16]. However, as salt is currently extremely cheap (e.g., wholesale prices of $0.7 per kg in NZ) the extra cost for salt from processed food would be under 1 cent per day (for the average NZ intake of 9 g salt per day) and so was ignored.
	It was considered out-of-scope given our health system perspective to consider reformulation costs and costs associated with package labelling changes. Our approach is also in accord with past NZ laws relating to food labelling, alcohol labelling and tobacco labelling in that manufacturers are not compensated for the costs imposed by the new law. For example, the NZ law requiring pictorial health warnings on tobacco packaging did not compensate industry for printing costs or lost sales. Furthermore, we assumed no additional costs from the existing routine evaluation efforts by the NZ Government (nutrition surveys and food surveys) and negligible enforcement and legal costs associated with non-compliance (owing to the relatively low levels of corruption in the NZ setting and the high compliance with laws e.g., the law banning smoking in bars and restaurants [69]).
Salt substitution at 25 %	As per the row above, i.e., the cost of a new law.
Tight limits on sodium in bread (280 mg/100 g)	The cost is just that of the cost of a new law for NZ (see above). As per the arguments above, there was no consideration of reformulation costs and package labelling costs. The technology exists to manufacture lower sodium bread as per examples already present on the NZ market e.g., 186 mg/100 g for one multigrain bread [30].
Modest limits on sodium in bread (400 mg/100 g)	As per the row above.

Of note is that gaps in Health Tracker data exist in specific areas (e.g., some private sector expenditure and the health-related aspects of residential care) and so we scaled up both the CVD disease costs and the annual health system costs for the non-diseased population. For the disease costs we scaled up Health Tracker costs across all age groups by 1.2, given that 83 % of all health sending in New Zealand is public (i.e., 1/0.83 = 1.2). Finally, costs at older ages were multiplied by 1.1, 1.2, 1.3 for the 65–74, 75–84 and 85+ age groups respectively to capture the estimated missing data of funding residential ‘disability support services’ care funded through government (‘Vote:Health’) but not yet captured in available data. All costs included those in the last six months of life.

#### Validation

Validation of model parameters and the final model outputs (relative to two official data sources) are detailed in an online Validation Report [32]. This additional work also involved parameter coherence checking, using the epidemiological software program DisMod II [33]. Subsequently, we also conducted a model validation exercise by comparing our TreeAge model with a multi-state life-table model, similar to the one used in a tobacco tax modelling study [34]. For the same sodium reduction intervention of a 22.8 mmol/day reduction in dietary intake (as used in our previous modelling [5]), the overall QALYs gained were 110,000 in our TreeAge model and 103,000 in the multi-state life-table model (both with 3 % discounting). We regarded this 6 % difference in results as acceptable given the models differed slightly in aspects of model structure and in baseline disease incidence rates and baseline case-fatality rates.

#### Analysis

For each of the interventions a reduction in dietary sodium intake was linked to a reduction in systolic blood pressure (BP) based on values derived from the regression models developed by Law et al [35]. A reduction in systolic BP was then linked to a reduced probability of adverse health outcomes (CHD and stroke) as per a meta-analysis of 61 prospective studies by Lewington et al [36] (for further details see Table 2). Analyses were by sex, ethnicity, age-group and time period in which the health gains were realised. We reran models (for expected values only) for a range of scenarios (e.g., the discount rate: at 0 and 6 %).

## Results

The largest health gains were from (in descending order): (i) a legal requirement to replace some of the sodium chloride in all processed foods with other salts at the 59 % level which is the maximal level from trial data; (ii) a lower level of salt substitution at the 25 % level; (iii) a legal requirement for a maximum level of sodium in bread of 280 mg/100 g; and (iv) a less stringent maximum level of sodium in bread of 400 mg/100 g. The salt substitution at the 59 % level produced a health gain of 294,000 QALYs over the lifetime of the cohort (95 % UI: 238,000 to 359,000), or an increase in the total discounted QALYs over the remainder of the 2011 cohort’s life of 0.89 % (i.e., 294,000/33,200,000). It also produced net cost-savings of NZ$ 1.5 billion (US$ 1.0 billion) (95 % UI: NZ$ 1.1 to 2.0 billion) (Table 5). All the other interventions were also cost-saving.

**Table 5:** Population level results for the costs and health gain of the four sodium reduction interventions (95 % uncertainty intervals in parentheses)^a^ and compared with previous interventions [5] using the same model (considered in the Discussion)

Modelled intervention	Health gain (QALYs for remainder of the cohort’s life)	Health system cost (NZ$; millions) for remainder of the cohort’s life	Incremental cost-effectiveness ratio (ICER)
“Do nothing” comparator^b^	33,200,000 (33,000,000 to 33,500,000)	162,000 (146,000 to 180,000)	-
1) Salt substitution at the 59 % level (processed food)	294,000 (238,000 to 359,000)	−1500 (−1980 to − 1090)	Dominant
2) “Sinking lid” for salt supply to the market^c^	211,000 (170,000 to 255,000)	−1110 (−1460 to − 830)	Dominant
3) “Salt tax”^c^	195,000 (159,000 to 237,000)	−1000 (−1320 to − 740)	Dominant
4) Salt substitution at 25 %	121,000 (97,300 to 147,000)	−620 (−820 to − 450)	Dominant
5) Mandatory 25 % reduction of sodium in all processed foods (“Mandatory-All”^c^)	110,000 (87,500 to 135,000)	−600 (−800 to − 440)	Dominant
6) UK Package (media campaign and voluntary action by industry)^c^	85,100 (69,600 to 102,000)	−440 (−570 to − 320)	Dominant
7) Mandatory 25 % reduction of sodium in bread, processed meats and sauces (“Mandatory-3G” ^c^)	61,700 (49,700 to 74,900)	−340 (−440 to − 240)	Dominant
8) Tight limits on sodium in bread (280 mg/100 g)	43,500 (34,700 to 52,800)	−220 (−290 to − 160)	Dominant
9) UK style “Mass Media Campaign”^c^	25,200 (14,200 to 36,700)	−120 (−200 to − 62)	Dominant
10) Modest limits on sodium in bread (400 mg/100 g)	15,600 (12,600 to 18,900)	−83 (−110 to − 61)	Dominant
11) Endorsement Label Programme^c^(current practice in NZ)	7900 (5500 to 10,400)	−34 (−52 to − 19)	Dominant
12) Dietary counselling by dietitians^c^(current practice in NZ)	200 (100 to 330)	6.90 (4.20 to 10.2)	NZ$36,900 per QALY gained

^a^Based on 2000 Monte Carlo simulations for the NZ adult population aged 35+ years and alive in 2011 modelled out to death or age 100. Discount rate: 3 %. Numbers are rounded to two or three meaningful digits

^b^No intervention costs are included in this “do nothing comparator” (i.e., the costs of the currently existing programmes of “dietary counselling by dietitians” and the “Endorsement Label Programme”[5] are removed)

^c^For further details see this previous work [5] but in summary: *Sinking Lid* the amount of food-grade salt released onto the NZ market is reduced annually to the point where the recommended level of sodium intake is achieved (2300 mg/d), *Salt Tax* an excise tax is applied and increased up to the point where the recommended level of sodium intake is achieved (2300 mg/d), *Mandatory-All* reduction of sodium in all processed foods by 25 % relative to existing levels, *UK Package* the mix of media campaign, voluntary food reformulation and food labelling changes used in the UK which resulted in a 15 % reduction in 24-h urinary sodium over 7 years in the adult population, *Mandatory-3G* mandatory reduction of sodium in the manufacture of breads, processed meats and sauces (by 25 % in each group), *UK Mass Media Campaign* just the mass media campaign part of the UK Package (detailed above), *Endorsement Label Programme* a programme involving an endorsement label run by the Heart Foundation (part of current practice in NZ), *Counselling* dietary counselling by dietitians to reduce sodium intake (part of current practice in NZ)

Overall cost results were largely driven by averted disease treatment costs for CVD, followed by the increased health system costs from extra life lived (as a result of the interventions), (Additional file 1: Table S1). Scenario analyses showed that even with a higher 6 % discount rate, all interventions still produced net health system cost-savings (Additional file 1: Table S2).

In terms of the impact for different demographic groups, for all the interventions the QALYs gained were higher and the cost-savings greater for younger age groups (<65 years) (Table 6). The same pattern existed for the greater health benefit and greater cost-saving for men compared to women (a 23 % extra health gain for men for the salt substitution at 59 % intervention). QALYs gained by Māori were also greater than for non-Māori e.g., by an extra 33 % for the salt substitution at 59 % (0.163 QALYs per adult vs 0.123 respectively).

**Table 6:** Net costs and QALYs incremental to “do nothing” by sociodemographic group for the four sodium reduction interventions (expressed per adult in 2011; discounted 3 %)

Intervention/population group	Incremental (to “do nothing”) cost per adult in NZ$	QALYs gained per adult
Salt substitution at 59 %		
Age < 65 years (starting in 2011)	−835	0.146
Age 65+ years (starting in 2011)	−112	0.073
Women	−550	0.115
Men	−760	0.141
Māori	−533	0.163
Non-Māori	−658	0.123
Salt substitution at 25 %		
Age < 65 years (starting in 2011)	−348	0.060
Age 65+ years (starting in 2011)	−46	0.030
Women	−230	0.048
Men	−316	0.058
Māori	−224	0.067
Non-Māori	−275	0.051
Tight limits on sodium in bread (280 mg/100 g)		
Age < 65 years (starting in 2011)	−125	0.022
Age 65+ years (starting in 2011)	−16	0.011
Women	−82	0.017
Men	−113	0.021
Māori	−80	0.024
Non-Māori	−98	0.018
Modest limits on sodium in bread (400 mg/100 g)		
Age < 65 years (starting in 2011)	−45	0.0072
Age 65+ years (starting in 2011)	−10	0.0054
Women	−30	0.0060
Men	−43	0.0075
Māori	−31	0.0087
Non-Māori	−37	0.0066

### Age and time distribution of the health gain

**Fig. 1:**
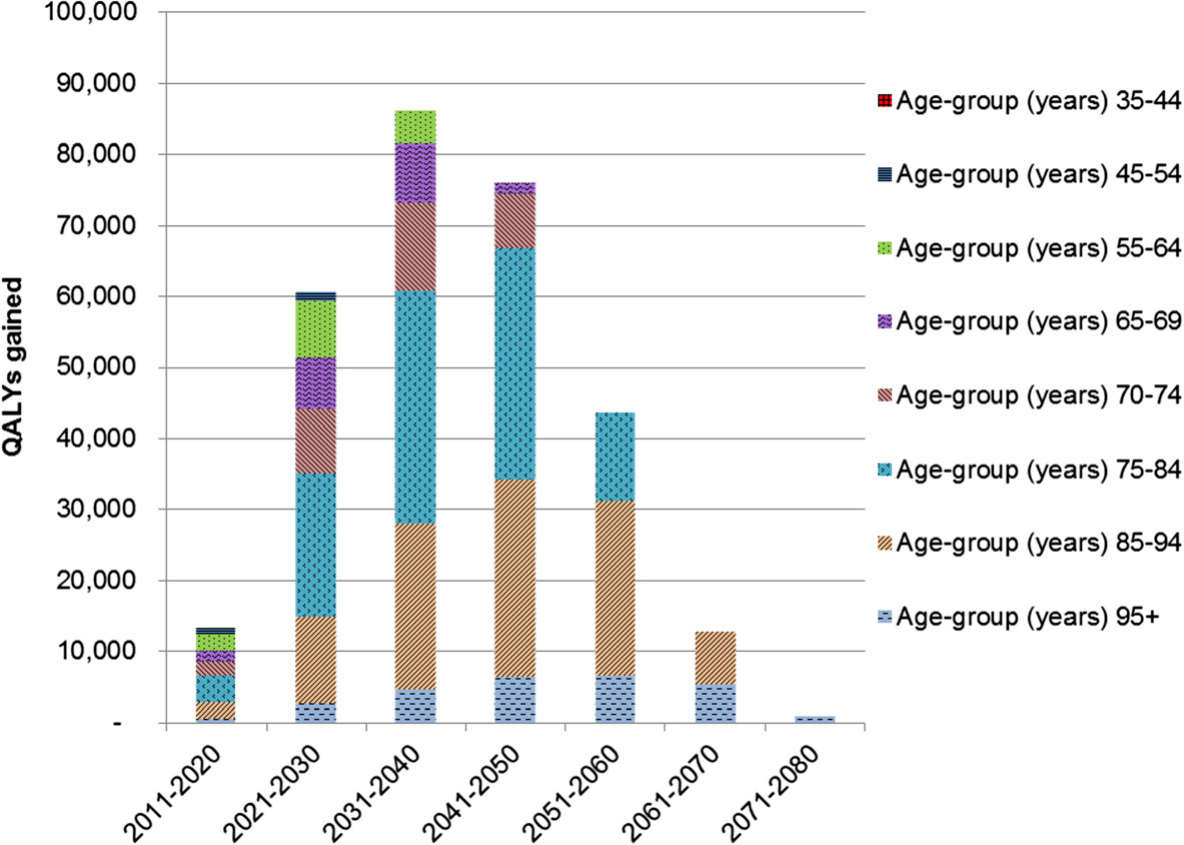
Ages at which the health gain occurs for the salt substitution (59 % level) intervention (discount rate of 3 %)

The distribution of the health gain by age-group and time is shown in Table 7 and Fig. 1 for the salt substitution (59 %) intervention. The age grouping here is the age at which the QALYs are gained, not the starting age in 2011 as in Table 6. Within the first 10 years after the intervention (i.e., 2011 to 2021), 4.6 % of all discounted QALYs that are estimated to arise over the remainder of the 2011 population’s life occur (or around 1300 QALYs per year in these first 10 years, out of 294,000 over the life course). Within the first 20 years of the intervention this proportion is 25.1 %.

**Table 7:** Age ranges in which the health gain occurs for the salt substitution intervention (at the 59 % level in processed food^a^, discount rate of 3 %)

Age when the QALYs are gained (i.e., not age in 2011)	In first 10 year period (i.e., 2011 to 2020)			In second 10 year period (i.e., 2021 to 2030)		
	QALYs gained	% of QALYs among 45+ year olds	% of QALYs among 55+ year olds	QALYs gained	% of QALYs among 45+ year olds	% of QALYs among 55+ year olds
35–44	51			-		
45–54	716	5.4 %		1,207	2.0 %	
55–64	2,220	16.9 %	17.8 %	8,016	13.2 %	13.5 %
65–69^b^	1,928	14.6 %	15.5 %	8,978	14.8 %	15.1 %
70–74	1,673	12.7 %	13.4 %	7,259	12.0 %	12.2 %
75–84	3,787	28.7 %	30.4 %	20,322	33.5 %	34.2 %
85–94	2,316	17.6 %	18.6 %	12,180	20.1 %	20.5 %
95+	537	4.1 %	4.3 %	2,695	4.4 %	4.5 %
Sum 45+	13,177	100 %		60,658	100 %	
Sum 55+	12,461		100 %	59,451		100 %

^a^See the Additional file 1 for the other three interventions

^b^The age at which welfare payments for all older people begin is 65 years in this New Zealand setting

By the age when QALYs accrue (i.e., the age in the future, not the age at 2011), 22.1 % of the QALY gains in the first 10 years accrue to less than 65 year olds. Similarly, 36.9 % accrue to less than 70 year olds. Very similar patterns in terms of proportions were seen for the other three interventions (Additional file 1).

## Discussion

### Main findings and interpretation

The estimated health gains from these modelled interventions were relatively large, particularly for the salt substitution intervention at the 59 % level. Indeed, this particular intervention generated more health gain and saved more health costs than any of the other eight sodium reduction interventions we have previously studied using the same model (Table 5). Such benefits from salt substitution would make this intervention particularly attractive in theory, but there is also field experience with the use of such substitutes having played a role in sodium intake reductions in Finland [28]. Large food companies (such as Unilever [37]) are also using now using potassium salts to replace sodium in their products.

The interventions around maximum levels of sodium in bread produced lower health gain than the salt substitution and other previously modelled interventions, but still generated more than the two “current practice” interventions in New Zealand (at the bottom of Table 5).

The interventions we studied were estimated to contribute to reducing health inequalities, by disproportionately benefiting both the health of the indigenous Māori population and men’s health. The greater per capita QALY gains for Māori are a direct function of the higher absolute background rates of CHD and stroke (given largely non-differential impact of the interventions). Our results are similar to a US study that found greater benefits for sodium reduction for Black compared to non-Black Americans [18]. But these results contrast to another US study that suggested no impact of sodium reduction in packaged and restaurant foods on ethnic inequalities [38]. Such variation suggests the value of country-specific studies where issues such as salt-sensitivity and levels of processed food consumption by demographic group can be specifically considered.

The majority of the modelled health gains from these salt reduction interventions occur beyond typical working ages and more than 20 years into the future (Fig. 1). Nevertheless, when considering just the first 10 years post-intervention, there is a reasonable benefit for the working-age population (<65 years) of 22 % of the QALY gains in this period (and 37 % for those aged <70) for the salt substitution intervention (at 59 %) (Table 7). This suggests that there is some justification for policy-makers considering the likely productivity gains among workers and especially for those citizens who continue to participate in the formal economy after reaching age 65 years (22 % for part or full-time employment as per the 2013 Census).

The collective results of this modelling have international applicability since high sodium intakes are a risk to health in virtually every country, and particularly in developed countries (where high sodium intake is the eighth most important risk factor for health loss [1]). But for some of the interventions there will of course be specific considerations around feasibility and effectiveness e.g., a relatively well-governed society might be needed to ensure compliance with laws around salt substitution and maximum levels of sodium in bread.

### Study limitations

As with all such modelling work there are limitations that should make policy-makers cautious in how they use any specific results, even if they can have reasonable confidence in the general pattern of results. These limitations include:

- Issues with model structure; and indeed our uncertainty estimates do not capture uncertainty arising from “model structure uncertainty”. For example, this model did not capture potential benefits of salt reduction on preventing stomach cancer [39] and renal disease [40]. Also the benefit of salt substitution was considered to be from the sodium reduction only and did not include any benefits to cardiovascular health arising from the addition of extra potassium (as might be expected as per this systematic review [41]). We also did not consider the benefit of salt substitution in terms of magnesium intake (e.g., the salt used in the trial in the Netherlands [27] used a natural salt from Iceland that was 17 % magnesium salts). Indeed, there is evidence that higher dietary intake of magnesium is associated with reduced CVD mortality according to a systematic review [42]. Similarly, for reduced risk of metabolic syndrome according to another systematic review [43]. Magnesium is also important for bone health [44] and yet median intakes of magnesium in North America are below the Recommended Daily Allowance [44]. Furthermore, a New Zealand study suggests that many women may also have suboptimal intakes given how higher magnesium intake is associated with having infants with higher birthweights [45]. But if any population wide salt substitute intervention did use magnesium-containing salts, then intakes of magnesium should ideally be monitored in ongoing nutrition surveys (to ensure that levels were in a safe range).
- While there is little scientific uncertainty that high levels of sodium intake are hazardous to health, the point at which dietary sodium reduction ceases to be beneficial is still debated (hence the wide 1000 mg to 5000 mg range for the “theoretical minimum risk exposure level” used in Global Burden of Disease Study 2013 [1]). But as per the collected evidence noted in the Introduction, it still seems reasonable to model benefits of sodium reductions toward the World Health Organization (WHO) target of 2000 mg sodium per day (5 g salt) in adults [4].
- Although New Zealand has relatively high quality epidemiological and costing data [24], these data still have limitations. For example, there are some limitations with estimating the precise prevalence of CVD [5] and around some of the health system costings [24].
- It is possible that for some processed foods the 59 % sodium substitution level might impact on palatability (though this was not the case in a randomised trial in the Netherlands [27] and in a study that achieved 67 % sodium reduction in bread using potassium chloride and yeast extract for flavour compensation [46]). However, concerns over palatability could conceivably result in the food industry adding more sugar to some processed foods (suggesting the need for careful monitoring by government agencies after any such interventions). Similarly, some people might respond to changes in perceived saltiness of food by adding more salt at the table.

### Potential research and policy implications

In this study, we have explored the potential benefits for health in working-age adults using a health system perspective. Further research using a societal perspective that would capture the economic benefits, such as increasing productivity at work, of preventing CVD in such people would be desirable. For example, one New Zealand study found that four years after a stroke in working age adults there was a 19 percentage point reduction in employment and a 15 % reduction in monthly person incomes [47].

Further lessons could also be drawn from the real-world data on the impact of down-regulating sodium in processed foods (e.g., as per recent laws in South Africa [48] and regulating levels of sodium in bread as adopted by various European countries [49]). A large randomised trial around salt substitution in China [15] is likely to be particularly important, albeit with some favourable findings around effectiveness and acceptability already published [14].

Research around packages of nutritional interventions is also highly desirable, whereby interventions such as a “salt tax” are part of a more general package of “junk food taxes” combined with subsidised school meals and subsidised fruit and vegetables. This might help avoid such responses as manufacturers adding more sugar to processed foods as sodium levels are down-regulated.

Even so, waiting for such additional research may not be prudent if policy-makers are concerned about reducing the health loss from NCDs in their countries and if their health systems are financially constrained. If so, they could start by considering the adoption of the interventions modelled internationally (Table 1) and by us (Table 5). They could also follow the specific salt reduction actions that have already been implemented by other nations [28, 49–51].

Policy-makers who still remain sceptical of the scientific basis for dietary sodium reduction and of the results of modelling studies, still have intervention options. They could focus on more comprehensive nutritional approaches such as “junk food taxes” as mentioned above, since these may impact on multiple nutritional hazards collectively (high sodium, high sugar, low polyunsaturated fatty acid levels, high energy density etc). Or for those policy-makers only concerned with reducing levels of sodium intake that virtually all researchers agree are hazardous (above the 1000 to 5000 mg sodium per day range as considered in the Global Burden of Disease 2013 Study [1]), then they could target interventions to products that may particularly contribute to such high intakes (e.g., high sodium breads, sauces and processed meats).

## Conclusions

This work modelled four dietary salt reduction interventions relating to salt substitution and regulating the maximum levels of sodium in bread. It found large health gain and cost-savings, particularly for the salt substitution interventions. Such health and cost-saving benefits are consistent with the international literature on sodium reduction interventions but this work provides additional evidence around salt substitution, the potential impact on working-age adults and on reducing health inequalities.

## Abbreviations

BP, blood pressure; CHD, coronary heart disease; CVD, cardiovascular disease; DR; discount rate; DW, disability weight; NCD, non-communicable disease; NZ, New Zealand; QALY, quality-adjusted life-year; UI, uncertainty interval; WHO, World Health Organization; YLD, year of life lived with disability.

## Additional file

Additional file 1: Additional Methods and Results. (DOCX 77 kb)

## References

[1] Forouzanfar M, Alexander L, Anderson H, Bachman V, Biryukov S, GBD 2013 Risk Factors Collaborators (2015). Global, regional, and national comparative risk assessment of 79 behavioural, environmental and occupational, and metabolic risks or clusters of risks in 188 countries, 1990-2013: a systematic analysis for the global burden of disease study 2013. Lancet.

[2] Beaglehole R, Bonita R, Horton R, Adams C, Alleyne G, Asaria P (2011). Priority actions for the non-communicable disease crisis. Lancet.

[3] Di Cesare M, Khang YH, Asaria P, Blakely T, Cowan MJ, Farzadfar F (2013). Inequalities in non-communicable diseases and effective responses. Lancet.

[4] WHO (2012). Guideline. Sodium intake for adults and children.

[5] Nghiem N, Blakely T, Cobiac LJ, Pearson AL, Wilson N (2015). Health and economic impacts of eight different dietary salt reduction interventions. PLoS One.

[6] He FJ, Li J, Macgregor GA (2013). Effect of longer-term modest salt reduction on blood pressure. Cochrane Database Syst Rev.

[7] Aburto NJ, Ziolkovska A, Hooper L, Elliott P, Cappuccio FP, Meerpohl JJ (2013). Effect of lower sodium intake on health: systematic review and meta-analyses. BMJ.

[8] Cook NR, Cutler JA, Obarzanek E, Buring JE, Rexrode KM, Kumanyika SK (2007). Long term effects of dietary sodium reduction on cardiovascular disease outcomes: observational follow-up of the trials of hypertension prevention (TOHP). BMJ.

[9] Cook NR, Appel LJ, Whelton PK (2014). Lower levels of sodium intake and reduced cardiovascular risk. Circulation.

[10] Chang HY, Hu YW, Yue CS, Wen YW, Yeh WT, Hsu LS (2006). Effect of potassium-enriched salt on cardiovascular mortality and medical expenses of elderly men. Am J Clin Nutr.

[11] Farquhar WB, Edwards DG, Jurkovitz CT, Weintraub WS (2015). Dietary sodium and health: more than just blood pressure. J Am Coll Cardiol.

[12] Siervo M, Lara J, Chowdhury S, Ashor A, Oggioni C, Mathers JC. Effects of the Dietary Approach to Stop Hypertension (DASH) diet on cardiovascular risk factors: a systematic review and meta-analysis. Br J Nutr. 2014;1–15. 10.1017/S0007114514003341 25430608

[13] Poggio R, Gutierrez L, Matta MG, Elorriaga N, Irazola V, Rubinstein A (2015). Daily sodium consumption and CVD mortality in the general population: systematic review and meta-analysis of prospective studies. Public Health Nutr.

[14] Li N, Prescott J, Wu Y, Barzi F, Yu X, Zhao L (2009). The effects of a reduced-sodium, high-potassium salt substitute on food taste and acceptability in rural northern China. Br J Nutr.

[15] Li N, Yan LL, Niu W, Labarthe D, Feng X, Shi J (2013). A large-scale cluster randomized trial to determine the effects of community-based dietary sodium reduction--the China Rural Health Initiative Sodium Reduction Study. Am Heart J.

[16] Peng YG, Li W, Wen XX, Li Y, Hu JH, Zhao LC (2014). Effects of salt substitutes on blood pressure: a meta-analysis of randomized controlled trials. Am J Clin Nutr.

[17] Martikainen JA, Soini EJ, Laaksonen DE, Niskanen L (2011). Health economic consequences of reducing salt intake and replacing saturated fat with polyunsaturated fat in the adult Finnish population: estimates based on the FINRISK and FINDIET studies. Eur J Clin Nutr.

[18] Bibbins-Domingo K, Chertow GM, Coxson PG, Moran A, Lightwood JM, Pletcher MJ (2010). Projected effect of dietary salt reductions on future cardiovascular disease. N Engl J Med.

[19] Nghiem N, Wilson N, Blakely T (2014). Technical background to the cardiovascular disease model used in the BODE^3^ programme.

[20] Ministry of Health (2013). Ways and means: a report on methodology from the New Zealand burden of disease, injury and risk study, 2006–2016.

[21] Woodward A, Blakely T (2014). The healthy country? a history of life and death in New Zealand.

[22] OECD (2013). New international comparisons of GDP and consumption based on purchasing power parities for the year 2011.

[23] Baltussen R, Adam T, Tan-Torres Edejer T, Hutubessy R, Acharya A, et al. Methods for generalized cost-effectiveness analysis. In: Tan-Torres Edejer T, Baltussen R, Adam T, Hutubessy R, Acharya A, et al., editors. Making choices in health: WHO guide to cost-effectiveness analysis. Geneva: World Health Organization; 2003.

[24] Blakely T, Atkinson J, Kvizhinadze G, Nghiem N, McLeod H, Davies A (2015). Updated New Zealand health system cost estimates from health events by sex, age and proximity to death: further improvements in the age of ‘big data’. N Z Med J.

[25] Salomon JA, Vos T, Hogan DR, Gagnon M, Naghavi M, Mokdad A (2012). Common values in assessing health outcomes from disease and injury: disability weights measurement study for the global burden of disease study 2010. Lancet.

[26] Blakely T, Woodward A. Living longer, living healthier? Latest Official Report on independent life expectancy in NZ. Public Health Expert [Blog] (31 August, 2015). Available at: https://blogs.otago.ac.nz/pubhealthexpert/2015/08/31/living-longer-living-healthier-latest-official-report-on-independent-life-expectancy-in-nz. Accessed 6 May 2016.

[27] Geleijnse JM, Witteman JC, Bak AA, den Breeijen JH, Grobbee DE (1994). Reduction in blood pressure with a low sodium, high potassium, high magnesium salt in older subjects with mild to moderate hypertension. BMJ.

[28] He FJ, Jenner KH, Macgregor GA (2010). WASH-world action on salt and health. Kidney Int.

[29] Dunford EK, Eyles H, Ni Mhurchu C, Webster JL, Neal BC (2011). Changes in the sodium content of bread in Australia and New Zealand between 2007 and 2010: implications for policy. Med J Aust.

[30] Woodward E, Eyles H, Ni Mhurchu C (2012). Key opportunities for sodium reduction in New Zealand processed foods. Aust N Z J Public Health.

[31] Butler M (2010). Salt reduction targets agreed.

[32] Nghiem N, Wilson N, Blakely T (2014). Validation issues relating to the cardiovascular disease model developed in the BODE^3^ programme.

[33] Barendregt J, Oortmarssen GJ, Vos T, Murray CJL (2003). A generic model for the assessment of disease epidemiology: the computational basis of DisMod II. Popul Health Metr.

[34] Blakely T, Cobiac LJ, Cleghorn CL, Pearson AL, van der Deen FS, Kvizhinadze G (2015). Health, health inequality, and cost impacts of annual increases in tobacco tax: Multistate life table modeling in New Zealand. PLoS Med.

[35] Law MR, Frost CD, Wald NJ (1991). By how much does dietary salt reduction lower blood-pressure? 1. Analysis of observational data among populations. BMJ.

[36] Lewington S, Clarke R, Qizilbash N, Peto R, Collins R (2002). Age-specific relevance of usual blood pressure to vascular mortality: a meta-analysis of individual data for one million adults in 61 prospective studies. Lancet.

[37] Unilever. Reducing salt (circa 2015). Available at: https://www.unilever.com/sustainable-living/what-matters-to-you/reducing-salt.html Accessed 6 May 2016.

[38] Choi SE, Brandeau ML, Basu S (2016). Expansion of the national salt reduction initiative: a mathematical model of benefits and risks of population-level sodium reduction. Med Decis Making.

[39] D'Elia L, Rossi G, Ippolito R, Cappuccio FP, Strazzullo P (2012). Habitual salt intake and risk of gastric cancer: a meta-analysis of prospective studies. Clin Nutr.

[40] Smyth A, O'Donnell MJ, Yusuf S, Clase CM, Teo KK, Canavan M (2014). Sodium intake and renal outcomes: A systematic review. Am J Hypertens.

[41] Aburto NJ, Hanson S, Gutierrez H, Hooper L, Elliott P, Cappuccio FP (2013). Effect of increased potassium intake on cardiovascular risk factors and disease: systematic review and meta-analyses. BMJ.

[42] Fang X, Liang C, Li M, Montgomery S, Fall K, Aaseth J (2016). Dose-response relationship between dietary magnesium intake and cardiovascular mortality: A systematic review and dose-based meta-regression analysis of prospective studies. J Trace Elem Med Biol.

[43] Sarrafzadegan N, Khosravi-Boroujeni H, Lotfizadeh M, Pourmogaddas A, Salehi-Abargouei A (2016). Magnesium status and the metabolic syndrome: A systematic review and meta-analysis. Nutr.

[44] Price CT, Langford JR, Liporace FA (2012). Essential nutrients for bone health and a review of their availability in the average North American diet. Open Orthop J.

[45] Watson PE, McDonald BW (2010). The association of maternal diet and dietary supplement intake in pregnant New Zealand women with infant birthweight. Eur J Clin Nutr.

[46] Bolhuis DP, Temme EH, Koeman FT, Noort MW, Kremer S, Janssen AM (2011). A salt reduction of 50% in bread does not decrease bread consumption or increase sodium intake by the choice of sandwich fillings. J Nutr.

[47] Dixon S (2015). The employment and income effects of eight chronic and acute health conditions (treasury working paper 15/15).

[48] Hofman K, Tollman S (2013). Population health in South Africa: a view from the salt mines. Lancet Global Health.

[49] European Commission (2012). Survey on members states implementation of the EU salt reduction framework: directorate-general health and consumers.

[50] He FJ, Brinsden HC, Macgregor GA (2014). Salt reduction in the United Kingdom: a successful experiment in public health. J Hum Hypertens.

[51] Pietinen P, Mannisto S, Valsta LM, Sarlio-Lahteenkorva S (2010). Nutrition policy in Finland. Public Health Nutr.

[52] Murray CJ, Lauer JA, Hutubessy RC, Niessen L, Tomijima N, Rodgers A (2003). Effectiveness and costs of interventions to lower systolic blood pressure and cholesterol: a global and regional analysis on reduction of cardiovascular-disease risk. Lancet.

[53] Palar K, Sturm R (2009). Potential societal savings from reduced sodium consumption in the U.S. adult population. Am J Health Promot.

[54] Smith-Spangler CM, Juusola JL, Enns EA, Owens DK, Garber AM (2010). Population strategies to decrease sodium intake and the burden of cardiovascular disease: a cost-effectiveness analysis. Ann Int Med.

[55] Cobiac LJ, Vos T, Veerman JL (2010). Cost-effectiveness of interventions to reduce dietary salt intake. Heart.

[56] Barton P, Andronis L, Briggs A, McPherson K, Capewell S (2011). Effectiveness and cost effectiveness of cardiovascular disease prevention in whole populations: modelling study. BMJ.

[57] Wang G, Bowman BA (2013). Recent economic evaluations of interventions to prevent cardiovascular disease by reducing sodium intake. Curr Atheroscler Rep.

[58] Cobiac LJ, Magnus A, Lim S, Barendregt JJ, Carter R, Vos T (2012). Which interventions offer best value for money in primary prevention of cardiovascular disease?. PLoS One.

[59] Dodhia H, Phillips K, Zannou MI, Airoldi M, Bevan G (2012). Modelling the impact on avoidable cardiovascular disease burden and costs of interventions to lower SBP in the England population. J Hypertens.

[60] McLean R, Williams S, Mann J, Parnell W (2011). How much salt are we eating? Estimates of New Zealand population sodium from the 2008/2009 Adult Nutrition Survey.

[61] McLean RM, Mann JI, Hoek J (2011). World Salt Awareness Week: more action needed in New Zealand. N Z Med J.

[62] Huang L, Crino M, Wu JH, Woodward M, Barzi F, Land MA, et al. Mean population salt intake estimated from 24-h urine samples and spot urine samples: a systematic review and meta-analysis. Int J Epidemiol. 2016. 10.1093/ije/dyv313 26796216

[63] Ministry of Health (2006). Food and Nutrition Monitoring Report 2006.

[64] McLean RM, Williams S, Mann JI, Miller JC, Parnell WR (2013). Blood pressure and hypertension in New Zealand: results from the 2008/09 Adult Nutrition Survey. N Z Med J.

[65] Law MR, Morris JK, Wald NJ (2009). Use of blood pressure lowering drugs in the prevention of cardiovascular disease: meta-analysis of 147 randomised trials in the context of expectations from prospective epidemiological studies. BMJ.

[66] Wilson N (2014). Background technical details on mandatory interventions used in sodium reduction modelling for cardiovascular disease prevention.

[67] Wilson N, Nghiem N, Foster R, Cobiac L, Blakely T (2012). Estimating the cost of new public health legislation. Bull World Health Organ.

[68] BODE3 Programme (2012). Results for the cost of making a new law, all in $NZ.

[69] Wilson N, Edwards R, Parry R (2011). A persisting secondhand smoke hazard in urban public places: results from fine particulate (PM2.5) air sampling. N Z Med J.

