# Supplementary material for: The health gains and cost savings of dietary salt reduction interventions, with equity and age distributional aspects
Source: BMC Public Health. 2016 May 23;16:423. doi: 10.1186/s12889-016-3102-1 (PMC4877955; doi:10.1186/s12889-016-3102-1)
Supplement: Additional file 1: — Additional Methods and Results. (DOCX 77 kb) [file 12889_2016_3102_MOESM1_ESM.docx]

## Appendix

## Additional Methods

As further context, New Zealand is a fairly typical OECD country in terms of health spending (at 10.0% of GDP – slightly more than the OECD average of 9.3%). But 83% of health spending was funded by public sources in 2011 (which is well above the average of 72% in OECD countries). Residential care for the elderly in New Zealand is largely funded from social welfare budget and so is excluded from our analysis – given the health system perspective being taken. Nevertheless, the residential care costs that relate specifically to health (i.e., residential care hospital facilities) is captured in our analyses (via scaling up from Health Tracker costs – see the Methods in the main manuscript).

Future trends in health costs were not modelled as these are considered very uncertain due to reasons around the New Zealand economy’s dependency on commodity prices, recent expansion in the role of the government’s pharmaceutical purchasing agency, and potential future trade agreements that might limit the government’s capacity to constrain health costs.

## Additional Results

Table A1: Types of costs (NZ$) by the four sodium reduction interventions (expressed per adult in 2011, discount rate of 3%)

| **Intervention** | **Direct intervention cost** | **CVD health system costs** | **Non-CVD health system costs** | **Net cost** |
| --- | --- | --- | --- | --- |
| ***Baseline*** |  |  |  |  |
| “Do nothing” comparator | – | 16,000 | 54,500 | 70,500 |
| Salt substitution at 59% | 1.43 | 14,600 | 55,200 | 69,800 |
| Salt substitution at 25% | 1.43 | 15,400 | 54,800 | 70,200 |
| Tight limits on sodium in bread | 1.43 | 15,800 | 54,600 | 70,400 |
| Modest limits on sodium in bread | 1.43 | 15,900 | 54,500 | 70,400 |
| ***Incremental to “Do Nothing” costs*** | |  |  |  |
| Salt substitution at 59% | 1.43 | -1310 | 660 | -650 |
| Salt substitution at 25% | 1.43 | -550 | 270 | -270 |
| Tight limits on sodium in bread | 1.43 | -200 | 98 | -97 |
| Modest limits on sodium in bread | 1.43 | -72 | 34 | -36 |

Table A2: Scenario analysis relating to the four sodium reduction interventions (cost, health gain and cost-effectiveness per individual New Zealand adult)

| **Scenarios** | **Incremental cost per adult (NZ$)** | **QALY gain per adult** | **ICER (cost per QALY)** |
| --- | --- | --- | --- |
| ***Salt substitution at 59%*** |  |  |  |
| 3% discount rate | -650.31 | 0.127 | Dominant |
| 0% discount rate | -702.63 | 0.317 | Dominant |
| 6% discount rate | -478.06 | 0.059 | Dominant |
| ***Salt substitution at 25%*** |  |  |  |
| 3% discount rate | -271.18 | 0.053 | Dominant |
| 0% discount rate | -294.29 | 0.130 | Dominant |
| 6% discount rate | -199.23 | 0.025 | Dominant |
| ***Tight limits on sodium in bread*** |  |  |  |
| 3% discount rate | -125.06 | 0.0154 | Dominant |
| 0% discount rate | -175.35 | 0.0397 | Dominant |
| 6% discount rate | -71.03 | 0.0088 | Dominant |
| ***Modest limits on sodium in bread*** |  |  |  |
| 3% discount rate | -35.93 | 0.0068 | Dominant |
| 0% discount rate | -37.11 | 0.0155 | Dominant |
| 6% discount rate | -28.34 | 0.0035 | Dominant |

Table A3. Age ranges in which the health gain occurs for all four salt interventions (discount rate of 3%)

| **Time period** | **Age when the QALYs are gained (i.e. not age in 2011)** | **Modest limits on sodium in bread (400mg/100g)** | | | **Tight limits on sodium in bread (280mg/100g)** | | | **Salt substitution at 25%** | | | **Salt substitution at 59%** | | |
| --- | --- | --- | --- | --- | --- | --- | --- | --- | --- | --- | --- | --- | --- |
|  |  | **QALYs gained** | **% of QALYs among 45+ year olds** | **% of QALYs among 55+ year olds** | **QALYs gained** | **% of QALYs among 45+ year olds** | **% of QALYs among 55+ year olds** | **QALYs gained** | **% of QALYs among 45+ year olds** | **% of QALYs among 55+ year olds** | **QALYs gained** | **% of QALYs among 45+ year olds** | **% of QALYs among 55+ year olds** |
| **In first 10 years (i.e., 2011 to 2020)** | 35-44 | 10 |  |  | 8 |  |  | 22 |  |  | 51 |  |  |
|  | 45-54 | 94 | 5.3% |  | 110 | 5.5% |  | 303 | 5.5% |  | 716 | 5.4% |  |
|  | 55-64 | 291 | 16.5% | 17.4% | 341 | 16.9% | 17.9% | 939 | 16.9% | 17.9% | 2,220 | 16.9% | 17.8% |
|  | 65-69 | 256 | 14.5% | 15.3% | 295 | 14.7% | 15.5% | 814 | 14.6% | 15.5% | 1,928 | 14.6% | 15.5% |
|  | 70-74 | 224 | 12.7% | 13.4% | 256 | 12.7% | 13.5% | 707 | 12.7% | 13.5% | 1,673 | 12.7% | 13.4% |
|  | 75-84 | 513 | 29.1% | 30.7% | 579 | 28.7% | 30.4% | 1,598 | 28.7% | 30.4% | 3,787 | 28.7% | 30.4% |
|  | 85-94 | 317 | 18.0% | 19.0% | 353 | 17.5% | 18.53% | 975 | 17.5% | 18.65% | 2,316 | 17.6% | 18.6% |
|  | 95+ | 69 | 3.9% | 4.1% | 81 | 4.0% | 4.3% | 224 | 4.0% | 4.3% | 537 | 4.1% | 4.3% |
|  | **Sum 45+** | **1,764** | **100%** |  | **2,016** | **100%** |  | **5,562** | **100%** |  | **13,177** | **100%** |  |
|  | **Sum 55+** | **1,670** |  | **100%** | **1,906** |  | **100%** | **5,258** |  | **100%** | **12,461** |  | **100%** |
| **In 2^nd^ 10 years (i.e., 2021 to 2030)** | 35-44 |  |  |  |  |  |  |  |  |  |  |  |  |
|  | 45-54 | 71 | 1.9% |  | 185 | 2.0% |  | 511 | 2.0% |  | - |  |  |
|  | 55-64 | 464 | 12.3% | 12.5% | 1,226 | 13.4% | 13.7% | 3,382 | 13.3% | 13.6% | 1,207 | 2.0% |  |
|  | 65-69 | 558 | 14.8% | 15.1% | 1,362 | 14.9% | 15.2% | 3,767 | 14.9% | 15.2% | 8,016 | 13.2% | 13.5% |
|  | 70-74 | 438 | 11.6% | 11.8% | 1,105 | 12.1% | 12.3% | 3,054 | 12.0% | 12.3% | 8,978 | 14.8% | 15.1% |
|  | 75-84 | 1,294 | 34.3% | 34.9% | 3,065 | 33.5% | 34.2% | 8,494 | 33.5% | 34.2% | 7,259 | 12.0% | 12.2% |
|  | 85-94 | 788 | 20.9% | 21.3% | 1,822 | 19.9% | 20.3% | 5,062 | 20.0% | 20.4% | 20,322 | 33.5% | 34.2% |
|  | 95+ | 162 | 4.3% | 4.4% | 393 | 4.3% | 4.4% | 1,102 | 4.3% | 4.4% | 12,180 | 20.1% | 20.5% |
|  | **Sum 45+** | **3,777** | **100%** |  | **9,158** | **100%** |  | **25,372** | **100%** |  | 2,695 | 4.4% | 4.5% |
|  | **Sum 55+** | **3,706** |  | **100%** | **8,972** |  | **100%** | **24,862** |  | **100%** | **60,658** | **100%** |  |
|  |  |  |  |  |  |  |  |  |  |  | **59,451** |  | **100%** |
